# Dr. D. R. Jennings

**Published:** 1897-12

**Authors:** 


### Dr. D. R. Jennings.

Died at his home in Cleveland, on Friday, October 29th, 1897, David Rawson Jennings, D. D. S., in the 57th year of his age.

Dr. Jennings had been in failing health for several years, and though at times the disease seemed to be checked, and a hope of entire relief was entertained, yet the disease was all the while making its insidious way to a fatal termination; the affection was of renal character.

Dr. Jennings was born in Ravenna, Ohio, January 27th, 1840. Though not a graduate of a literary institution he had the advantage of Twinsburg Academy and of Knox College at Galesburg, Ills., and though he did not take the final course in either institution, he made the most of his opportunities. He also took one course in Starling Medical College, at Columbus, after which he studied dentistry with Dr. J. G. Willis of Ravenna, O. In 1862 he opened an office in that place, and there remained in the practice of his profession until 1872. In 1867 the degree of Doctor of Dental Surgery (honorary), was conferred upon him by the Ohio College of Dental Surgery. In 1872 he removed to Cleveland, and there established himself in practice and attained eminent success. In 1887 the M. D. degree was conferred on him by the Medical Department of Wooster University. He was a member of the leading dental societies of the country, and held positions of trust and honor in them.

He was regarded by the profession as one of its best operators and practitioners. He was a man of sterling integrity, and had a high sense of honor, and a supreme contempt of all sorts of quackery. He was a member of high standing in some of the leading orders of the country, and has been honored with their highest positions. In 1861 he married Miss Elizabeth Monroe, two children were born to them, William M. in 1866, and Andrew R. in 1870.

The immediate occasion of Dr. Jennings death is more than usually sad, because it was by his own hand. The disease under which he had so long been afflicted, had so unbalanced his mind that he could not be regarded as responsible for his acts.

Though Dr. Jenning's affliction was pretty well known to his friends, yet his death was a severe shock to all. The removal of such men is a great loss to the profession, and one it can ill afford to have. His life companion and the two sons will have the warmest sympathy of all who knew their loved one.

